# The expression pattern of Immune checkpoints after chemo/radiotherapy in the tumor microenvironment

**DOI:** 10.3389/fimmu.2022.938063

**Published:** 2022-07-28

**Authors:** Hamidreza Hassanian, Zahra Asadzadeh, Amir Baghbanzadeh, Afshin Derakhshani, Antoine Dufour, Nazanin Rostami Khosroshahi, Souzan Najafi, Oronzo Brunetti, Nicola Silvestris, Behzad Baradaran

**Affiliations:** ^1^Immunology Research Center, Tabriz University of Medical Sciences, Tabriz, Iran; ^2^Student Research Committee, Tabriz University of Medical Sciences, Tabriz, Iran; ^3^Department of Biochemistry and Molecular Biology, University of Calgary, Calgary, AB, Canada; ^4^McCaig Insitute, Hotchkiss Brain Institute, and Snyder Institute for Chronic Diseases, University of Calgary, Calgary, AB, Canada; ^5^Departments of Physiology and Pharmacology, University of Calgary, Calgary, AB, Canada; ^6^Medical Oncology Unit, IRCCS Istituto Tumori Giovanni Paolo II, Bari, Italy; ^7^Medical Oncology Unit, Department of Human Pathology “G. Barresi” University of Messina, Messina, Italy; ^8^Department of Immunology, School of Medicine, Tabriz University of Medical Sciences, Tabriz, Iran

**Keywords:** cancer therapy, tumor microenvironment, chemo(radio)therapy, immune checkpoints, combination therapy

## Abstract

As a disease with the highest disease-associated burden worldwide, cancer has been the main subject of a considerable proportion of medical research in recent years, intending to find more effective therapeutic approaches with fewer side effects. Combining conventional methods with newer biologically based treatments such as immunotherapy can be a promising approach to treating different tumors. The concept of “cancer immunoediting” that occurs in the field of the tumor microenvironment (TME) is the aspect of cancer therapy that has not been at the center of attention. One group of the role players of the so-called immunoediting process are the immune checkpoint molecules that exert either co-stimulatory or co-inhibitory effects in the anti-tumor immunity of the host. It involves alterations in a wide variety of immunologic pathways. Recent studies have proven that conventional cancer therapies, such as chemotherapy, radiotherapy, or a combination of them, i.e., chemoradiotherapy, alter the “immune compartment” of the TME. The mentioned changes encompass a wide range of variations, including the changes in the density and immunologic type of the tumor-infiltrating lymphocytes (TILs) and the alterations in the expression patterns of the different immune checkpoints. These rearrangements can have either anti-tumor immunity empowering or immune attenuating sequels. Thus, recognizing the consequences of various chemo(radio)therapeutic regimens in the TME seems to be of great significance in the evolution of therapeutic approaches. Therefore, the present review intends to summarize how chemo(radio)therapy affects the TME and specifically some of the most important, well-known immune checkpoints’ expressions according to the recent studies in this field.

## Introduction

Cancer is the second-most common etiology of death worldwide after cardiac disease (1). Cancers cause the most disease-associated burden among different diseases all around the world, which is about 244.6 million Disability-Adjusted Life Years (DALYs), even more than ischemic heart disease (IHD) (2). Despite significant improvements in cancer therapy, it is still one of the leading health issues. So the explorations to find different solutions for this problem are ongoing. Our immune system combats cancer through various mechanisms involving different types of immune cells and molecules, such as cytokines and immune checkpoints. Malignant tumor cells use a wide variety of mechanisms to avoid and attenuate the immune system, which leads to uncontrolled proliferation of the cells, invasion and metastasis of the tumor, and at last, morbidity and mortality of cancer (3). The field of this battle between the host’s immune system and the tumor is known as the tumor microenvironment (TME), which is composed of different compartments such as the tumor and immune parts (4, 5). The tumor cells form and modulate the TME and dominate other components such as infiltrated immune cells and molecules (6). Immunotherapy is a relatively novel method of cancer therapy compared to conventional therapies such as chemo(radio)therapy. It acts by blocking the function of inhibitory immune checkpoints present on the various types of malignant and immune cells in the TME (7). Several studies have proven the efficacy of immunotherapy in treating different cancers. We can point to studies on various types of malignancies, including melanoma (8), non-small cell lung carcinoma (NSCLC) (9), head and neck malignancies (10), urinary tract cancers (11), colorectal carcinoma (CRC) (12), hepatocellular carcinoma (HCC) (13), Merkel cell carcinoma (14), and Hodgkin lymphoma (15). However, significant responses to immunotherapy are currently just seen in a limited number of cancers and patients. It indicates a need for searching for and designing more novel therapeutic strategies (16). One of these recently described novel approaches is the concept of “combination therapy.”

In this approach, we benefit from two or more mechanistically different methods such as immunotherapy and chemo(radio)therapy or surgery to induce synergistic, additive, and more robust attacks combating cancer (17–20). Combining conventional chemo(radio)therapeutic methods with immunotherapy seems to be one of the promising approaches. The TME characteristics differ widely across different types of cancers. Several studies have shown that various chemo(radio)therapy regimens alter the TME. The quality and pattern of these changes are associated with the type of tumor and the agents used during treatment (21). To design more effective combination therapies, we need to become more familiar with the exact properties of the TME across different tumors and with the changes induced by the chemo(radio)therapy. Many studies have demonstrated the alterations in the expression patterns of the immune checkpoints, as the crucial immunomodulatory molecules in the TME, in response to different chemo(radio)therapeutic regimens (22). Increasing our knowledge about the exclusive characteristics of the immune checkpoints, their mechanism(s) of function, and the related molecular pathways can help us design more efficient blocking agents. These immune-checkpoint inhibitors (ICIs) can be utilized as complementary therapy based on the changes caused by the conventional approaches, specifically chemo(radio)therapy. In the current study, we have reviewed the detailed properties of the TME and mentioned the bilateral role of the immune checkpoints in immune system-tumor interactions. Also, we evaluated the studies that assessed the changes caused by adjuvant and neoadjuvant chemo(radio)therapeutic therapies in the expression patterns of clinically valuable immune checkpoints.

## Tumor microenvironment - a key player in the immunoediting process and anti-tumor immunity

The concept of immune surveillance is the process of removing cancerous cells by the immune system based on recognizing specifically expressed neoantigens and stress-induced molecules in tumor cells. Lewis Thomas described this concept clearly and experimentally in the late 1950s for the first time (23, 24). Cancer immunoediting is a relatively new and more comprehensive concept, comprised of three phases: elimination phase (involving immune surveillance), equilibrium phase, and escape phase (3). Immune cells and factors put as much pressure as possible on tumor cells that survived the previous stage in the equilibrium phase. A significant population of cancerous cells is destroyed in this course, while a proportion develops new mutations making them resistant to the immune system’s attack. In the final escape phase, tumor variants that have become unsusceptible to the immune attacks extend in an unrestrained pattern (25). As a result, immunologically carved tumors expand steadily and become clinically evident (26). A wide variety of mechanisms altogether lead to the formation of tumor escape. These include decreased immune recognition by losing strong neoantigens, MHC class I, and co-stimulatory molecules. The other mechanism is increased resistance to cellular death by overexpression of anti-apoptotic molecules like Bcl-2. Tumors form an immunosuppressive tumor microenvironment (TME) by secreting cytokines like TGF-β and overexpressing co-inhibitory immune checkpoints such as Programmed Cell Death Protein 1(PD-1, CD279)/Programmed Death-ligand 1 (PD-L1), T cell Immunoglobulin domain, and Mucin domain 3 (TIM-3, CD366)/Galectin9, and Lymphocyte Activation Gene 3 (LAG-3) (27, 28).

**Figure 1:**
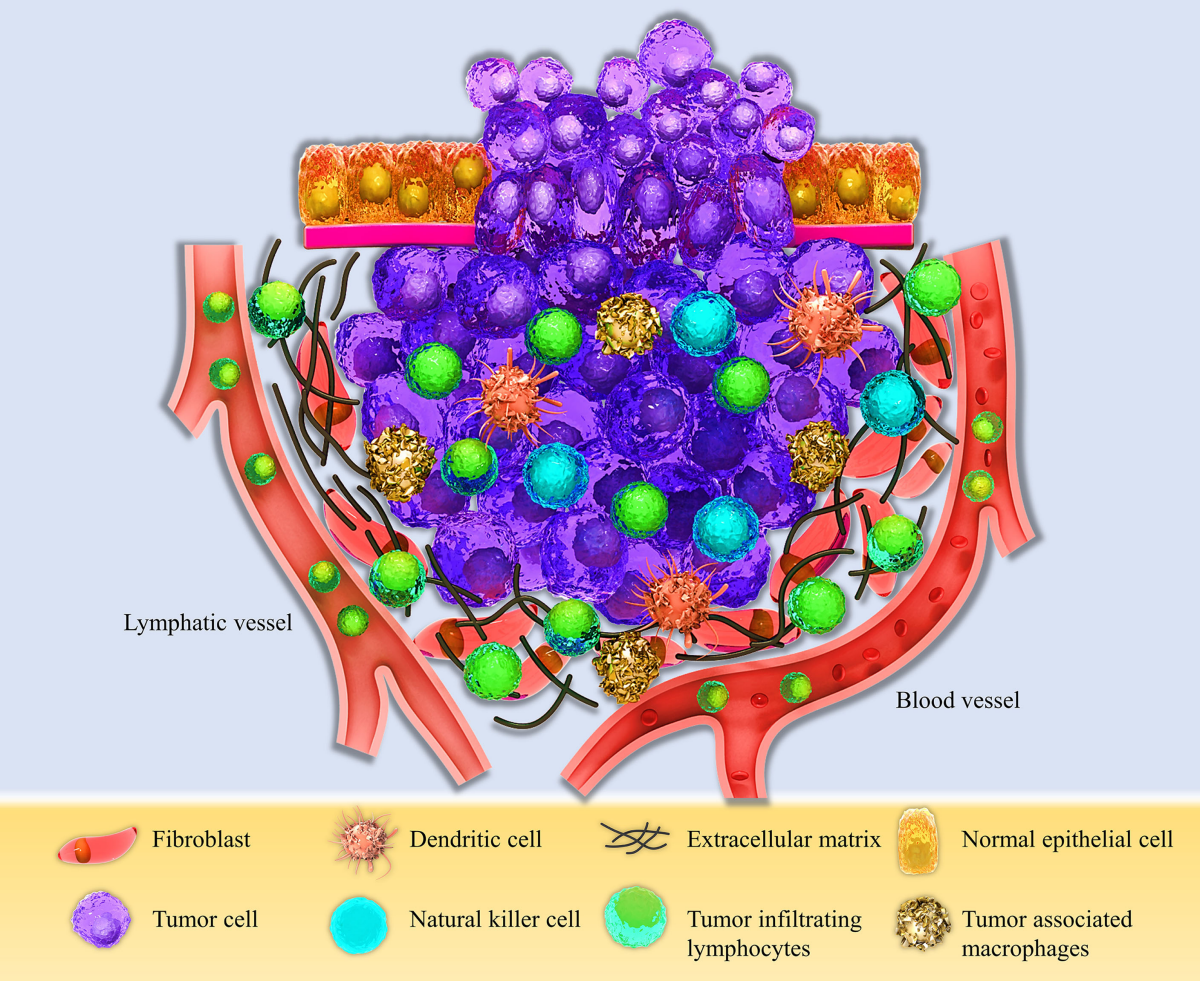
Schematic view of the Tumor microenvironment (TME). The TME consists of different compartments, including the proliferating tumor cells, tumor site, and tumor-infiltrating immune cells, such as Dendritic cells (DCs), Natural killer cells (NK cells), Tumor-associated macrophages (TAMs), and Tumor-infiltrating lymphocytes (TILs), and the stromal part containing fibroblasts, extracellular matrix, and lymphatic and blood vessels.

The tumor microenvironment is a unique environment that arises in the context of tumor progression due to tumor-host interactions. It is composed of different elements such as proliferating tumor cells, tumor stroma, infiltrating immune cells, blood vessels, and related tissue cells (**Figure 1**). TME is constructed, reformed, and controlled by the tumor at all times and has dominance over molecular and cellular events happening in neighboring tissues (25). Types of immune cells from both innate and adaptive parts are present in the TME (29). Natural killer (NK) cells are the innate immune system’s main effectors, constituting the first line of defense against tumors (30). Despite their ability to kill circulating cancerous cells, NK cell’s significance for battling and destroying established solid tumors seems to be unsure in the result of several mechanisms compromising their capacity to eliminate solid tumor cells, such as their inability to penetrate the core of the tumor and various immunoediting events leading to tumor escape (31). Research assessing immunophenotypes of several types of solid tumors in a wide population of patients with different types of cancers has shown some evidence of a T-cell infiltrated phenotype (32–34). Tumor-infiltrating lymphocytes (TILs) with various CD4^+^ to CD8^+^ T cells proportions build up a major part of TME. CD8^+^ cytotoxic T lymphocytes (CTLs) have been historically considered the pivotal cells in the immune system’s battle against tumors because of their ability to detect MHC class I mediated presentation of the intracellular antigens, expressed by all tumor cell types (35). CD4^+^ T helper cells (Th) also have a crucial role in immune defense against malignancies by various mechanisms such as activating antigen-specific effector cells and alarming innate immune cells such as macrophages, mast cells, and eosinophils (36, 37). These cells are activated in two main ways, directly by MHC class II expressing tumor cells and indirectly by antigen-presenting cells (APCs) present at the TME, such as dendritic cells (DCs) (38). Antigen-primed Th cells can directly activate tumor-antigen-specific CTLs through different routes such as direct interaction, improving CTL activity by co-stimulatory molecules on the surface of CTL, like CD127, CD34, and MHC class II, and enhancing CTL growth by secreting cytokines such as IL-12 (39).

In conditions associated with chronic inflammation like cancer and chronic infection, persistent antigen presentation and stimulation of T cell receptor (TCR) leading to activation of CTLs results in a gradual decrease in the effector activity of CTLs that finally disturbs response to tumors and infections. This phenomenon is called exhaustion (40–42). In this process, inhibitory molecules such as PD-1, Cytotoxic T lymphocyte Antigen-4 (CTLA-4, CD152), LAG-3, TIM-3, CD160, and T cell Immunoreceptor with Ig and ITIM domain (TIGIT) are significantly overexpressed in exhausted T cells, so they do not respond properly to the stimulation of TCR by presented antigens (43, 44). Exhausted CD8+ T cells do not proliferate well because they have impaired killing activity and secrete Low levels of effector cytokines such as INF-γ and TNF-α (45). The other subsets of T cells in TME are regulatory T cells (Treg) expressing the Foxp3 (Forkhead Box P3) molecule as their primary marker. These cells play a central role in stabilizing immune homeostasis and preventing autoimmunity (46). Considering their ability to avoid self-antigen responses, they may restrict anti-tumor immune response by different mechanisms such as activating inhibitory molecules mentioned before, like CTLA-4 (47–49). Plenty of studies have shown considerable infiltration of Treg cells into different types of tumors, such as in the head and neck, breast, lung, gastrointestinal tract, liver, pancreas, and ovary. On this basis, depleting TME from Tregs or manipulating their function in a specific manner can experimentally induce efficacious tumor immunity (50). Basic science findings clarifying the molecular and cellular mechanisms involved in T cell biology, as the facts mentioned above, have given rise to new therapeutic approaches toward malignancies, including immune checkpoint blocking by immunotherapy (51).

## Immune-checkpoints: A wide variety of molecules with a bilateral role in tumor-immune system battle

**Figure 2:**
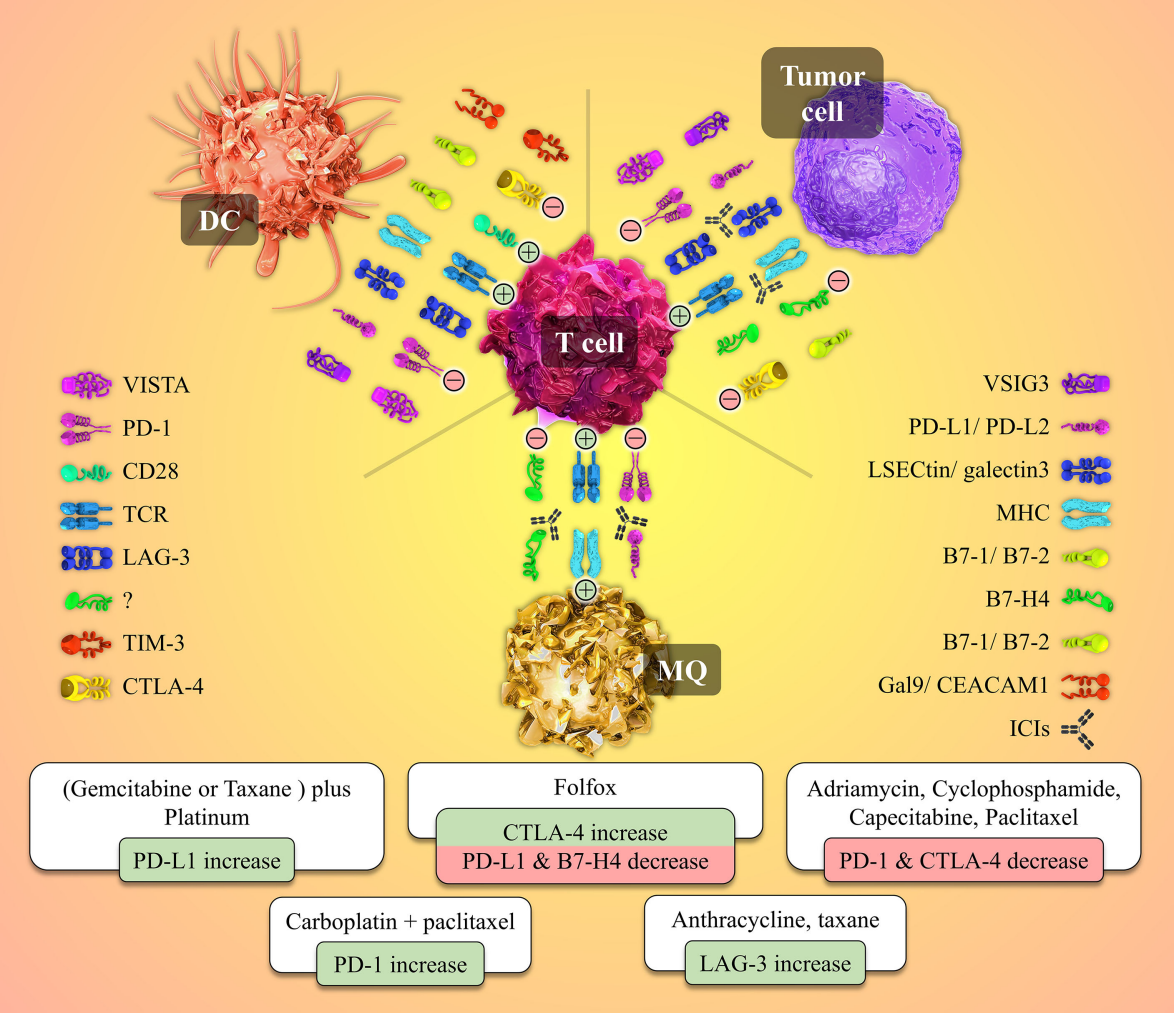
Cell to cell interactions and the role of the immune-checkpoint molecules and their receptors in the tumor microenvironment besides the patterns of immune-checkpoints expression patterns’ changes post-NAC with different chemotherapeutic agents. Tumor site T-cells need two activating signals to defend against and kill the tumor cells (shown by plus mark in a circle). The first signal is provided by the interaction between the T cell receptor (TCR) and its specific antigen presented by the MHC molecule on the Antigen-presenting cells (APCs) or the tumor cell. The second signal is a co-stimulatory one originating from CD28 and B7-1/B7-2 molecules interaction. Tumor cells overexpress inhibitory immune checkpoints to produce inhibitory signals and neutralize the positive ones (shown by the negative mark in a circle). Immune checkpoint inhibitors (ICIs), as a wide variety of drugs used in the immunotherapy of cancers, block the mentioned co-inhibitory function of the checkpoint molecules. Different chemotherapeutic agents alter the expression patterns of immune-checkpoint molecules by whether down-regulating or up-regulating the expression of these immune markers.

An appropriately working immune system protects the body from foreign pathogens and developing malignant tumors (52). Activation of immune effectors such as T cells is tightly controlled to prevent malfunctions such as dysregulations leading to autoimmunity. T lymphocytes need at least two stimulatory signals to be activated. The first signal is provided when the T cell receptor (TCR) recognizes the specific antigen the MHC molecule presents. A co-stimulatory signal is also needed to activate the T cell fully. For instance, CD80 or CD86 molecules on the surface of APCs interact with the CD28 molecule on the T cell and give rise to the co-stimulatory signal (53, 54). Also, in addition to immune checkpoints, different kinds of cytokines play crucial roles in this process (**Figure 2**).

Immune checkpoint receptors are in the membrane of various immune cells, mainly T cells and NK cells. When these cells face the specific antigens and previously mentioned ligands on the APCs, such as macrophages and DCs or the cancerous cells, they induce some signals which can be positive and stimulatory or negative and inhibitory. These signals originate from the interaction between these immune checkpoint receptors on the target cells and their ligands, i.e., checkpoint molecules on the effector cells. These negative and positive regulations exerted by the immune checkpoints and their receptors play a crucial role in stabilizing immune balance and homeostasis in the normal physiologic condition (55, 56).

Molecules such as CTLA-4, PD-1, TIM-3, TIGITT, and LAG-3 are checkpoint receptors with an immunosuppressive role. They generate inhibitory signals that avoid the full activation of effector cells, such as the CTLs. So, in the tumor-immune system battle, these molecules lead to immune exhaustion and provide a mechanism for the immune evasion of the tumors, suppressing the immune system’s anti-tumor potentiality (57, 58). Among checkpoint receptors with a co-stimulatory function are glucocorticoid-induced TNFR-related protein (GITR), CD 27, CD40, and OX40 from the superfamily of tumor necrosis factor receptors (TNFR). CD28 and inducible T cell co-stimulator (ICOS) are also stimulatory checkpoint receptors belonging to the B7-CD28 superfamily (59). The incompetent function of these molecules in the effector T cells recognizing the neoantigens expressed by the malignant cells in the TME weakens the anti-tumor immune response providing another opportunity for the immune evasion of the tumors. The concept of immunotherapy is based on the knowledge gained through the years about the two categories of checkpoint receptors and their ligands mentioned above. Based on these two groups of immune checkpoint functions, i.e., their either co-inhibitory or co-stimulatory function, there exist two approaches toward modulating and boosting the immune system to defend against malignancies more efficiently. One approach is to inhibit and antagonize the inhibitory checkpoints to prevent T cell exhaustion. It neutralizes the immunosuppressive effects. Immune checkpoint inhibitors (ICIs) are monoclonal antibodies (mAbs) developed against various classical and recently discovered inhibitory immune checkpoints (**Figure 2**) (60–62). Some of these ICIs are FDA-approved and widely used in treating different kinds of tumors such as melanoma, small and non-small-cell lung cancers, renal cell carcinoma, and gastric cancers (63).

The second approach in immunotherapy is based on the concept that augmenting the stimulatory functions of co-stimulatory immune checkpoints can empower the effector cells such as CTLs in the TME. It leads to more effective killing of the tumor cells. It is achievable by designing and utilizing agonistic antibodies that improve the positive signaling of these checkpoint molecules. It leads to more effective immune responses against malignancies (55).

The second approach has more extensive effects on the T cells than the first approach. It originates from different types of tumors expressing inhibitory immune checkpoints in different patterns. So, inhibition of a particular checkpoint molecule by the specific ICI is only beneficial when the targeted tumor significantly expresses that molecule at high levels (55). Despite broader effects, the second approach is accompanied by more risk of dangerous adverse effects such as multiple organ failure due to cytokine storm caused by CD28 activating antibody, theralizumab (64). This phenomenon has restricted the clinical use of this approach.

There are some cardinal problems with using ICIs. The first issue is that the quantity of the T cells present in the TME is a restricting factor. Low numbers of the TILs in the tumor compartment of the TME weakens the response of the tumor to the ICIs. The other problem is the adaptation of cancerous cells to a specific ICI by upregulating other co-inhibitory immune checkpoints that preserves the negative signals and avoids reversing TILs exhaustion. The latter problem can be solved by designing and utilizing bispecific antibodies (bsAb) that target two checkpoints simultaneously. Some of these bsAbs are in the market now (65, 66).

To solve the first problem, i.e., low numbers of TILs in the tumor site, the immunogenicity of the cancerous cells should be improved. Prompting immunologically mediated tumor cell death by taking advantage of cytotoxic methods or procedures targeting specific immune molecules can increase immunogenicity. The approach to combining other biological and non-biological therapies with immune checkpoint inhibition by ICIs to improve its efficacy is indeed the so-called “combination therapies.” Among non-biological therapeutic procedures are surgery, chemotherapy, and radiotherapy (67–69). Anti-cancer vaccines, antibodies against cytokines, oncolytic virotherapy, natural or synthetic cytokines, and chimeric antigen receptor (CAR) T cells are biological methods used in combination with ICIs (70–74). According to all we mentioned above, it is clear that cancer treatment is now multidimensional. Combining conventional approaches such as chemotherapy, radiotherapy, and surgery with relatively new therapies like immunotherapy and other biological methods may help us achieve better results by positively modifying the prognosis of different types of cancers (38). We need to improve our knowledge about the induced alterations in the TME of various tumors in response to adjuvant therapies. It helps us choose the most effective adjuvant therapies as different regimens used in the chemo(radio)therapy of cancers have diverse effects. Also, it can lead us to design more effective and specific combination therapies consisting of immunotherapy and conventional therapies. It is the first goal of this study. In the ongoing parts, we have reviewed the history, expression distribution, function, and changes in expression of some of the most important and clinically targeted immune checkpoints.

## PD-1

Programmed death-1 (PD-1) molecule, as a member of the immunoglobulin gene superfamily, was first discovered in 1992 (75). PD-1 is expressed on the surface of particular subsets of T cells and also non-T cell subsets like B cells and NK cells (**Table 1**) (16). This co-inhibitory immune checkpoint has a crucial role in stabilizing peripheral immune tolerance. For example, its knockout in C57BL/6 mice leads to an autoimmune pathology resembling what occurs in lupus erythematosus pathogenesis (76). PD-1, despite its name, has no role in the cell death induction process/apoptosis (47). This immune checkpoint has two ligands, PD-ligand-1 (PD-L1), expressed by a wide variety of somatic cells in response to pro-inflammatory cytokines, and PD-L2 (CD273 or B7-DC), with more restricted antigen-presenting expression (77). Activation of the PD-1 signaling pathway gives rise to transcriptional and epigenetics alterations in T cells, which finally leads to a decrease in the production of proteins such as inflammatory cytokines and finally T cell “exhaustion” in the TME (78).

**Table 1:** A summary of the immune checkpoints and their expression changes pattern in response to chemo/radio therapy.

Immune checkpoint molecules	Ligands	Distribution of the receptors	Function	Immune checkpoint inhibitor (ICI) drugs and some of the related clinical trials on ICIs.	The dominant pattern of expression changes post-NAC(R)
**PD-1**	PD-L1 (CD272) PD-L2 (CD273)	T cell subsets (TILs, Tregs, Effector T cells) Non-T cell subsets (NK cells, B cells, subsets of DCs)	Co-inhibitory effect by the PD-1/PD-L1 signaling pathway	Nivolumab (NCT01721759), Pembrolizumab (NCT02256436), Cemiplimab (NCT03002376)	A significant increase in expression levels was seen in most of the studies
**PD-L1**	PD-1 B7-1 (CD80)	T cells, B cells, NK cells, Monocytes, DCs	Co-inhibitory effect by the PD-1/PD-L1 signaling pathway	Durvalumab (NCT02639065), Avelumab (NCT03704467), Atezolizumab (NCT02425891)	A significant increase in expression levels was seen in most of the studies
**CTLA-4**	B7-1 (CD80) B7-2 (CD86)	Tregs Activated T cells	Co-inhibitory function by reducing IL-2 production, inhibiting T-cell proliferation, and eliminating B7-1,2 on APCs	Ipilimumab (NCT02279732), Tremelimumab (NCT01853618)	Opposing results in expression level alterations were seen, indicating a need for more studies
**LAG-3 (CD233)**	MHC-II, LSEctin, Galectin-3, FGLP-1	TILs, NK cells, B cells, DCs	Inhibitory regulatory effect on T-cell proliferation and DC activation	Eftilagimod alpha (NCT00349934), Relatlimab (NCT04611126), LAG525 (NCT03499899), MK4280 (NCT03598608), Sym022 (NCT03489369), REGN3767 (NCT03005782), TSR-033 (NCT02817633)	An increase in the expression levels was detected in the studies, but the number of studies was limited
**B7-H4**	B7-H4 receptor (Not well-known)	Cancerous cells (as in ovarian, uterus, and lung tumors), TAMs	Inhibitory function on activated effector T cells by decreasing IL-2 production and inducing cell-cycle arrest	FPA-150 (alsevalimab) (currently in phase Ia/Ib of the clinical trial in solid tumors,NCT03514121)	A single study demonstrated a decrease in expression levels that was associated with a better prognosis

The table represents a summary of the characteristics of the immune checkpoints, some of the ICI drugs, and related clinical trials evaluating their efficacy. Also, the dominant pattern of immune checkpoints’ expression changes in response to chemo/radio therapy due to the studies mentioned in previous sections on each immune checkpoint is presented.

Considering the PD-1/PD-L1 axis and its role in T cell anergy, several monoclonal antibodies have been designed to target these immune checkpoints. Some are FDA-approved, such as durvalumab, nivolumab, and pembrolizumab, which are currently used in immunotherapy of several types of cancers (**Table 1**) (60, 79–82). For example, CheckMate063, a phase2, single-arm trial, proved the activity and safety of nivolumab for patients with advanced, refractory NSCLC (83). Another clinical trial study demonstrated a reduced rate of death in advanced urothelial carcinoma patients with disease progression, during or following chemotherapy, as a result of treatment with pembrolizumab (Hazard Ratio (HR) = 0.73) (84). Realizing changes in PD-1 expression in the TME in response to chemo(radio)therapy across different types of tumors may help us design better combination therapies for managing the cancers. In recent years a limited number of studies have done this (**Table 1**).

According to a systematic review by Van den Ende et al., eight studies had assessed alterations in PD-1 expression patterns in response to chemo(radio)therapy until January 2019. Seven of these studies compared the level of PD-1 expression in the TME of pre-treatment to a post-treatment tissue. Also, one of them compared treated vs. untreated groups of a cohort. A total of five of these studies had statistically significant results. A significant increase in PD-1 expression was seen in four single studies in patients with ovarian cancer, breast cancer, non-small cell lung carcinoma (NSCLC), and glioblastoma. Also, a significant decrease after treatment was observed in a study on patients with breast cancer (85). In the latter study, breast tumor specimens of 33 women were evaluated immunohistochemically before and after neoadjuvant chemotherapy (NAC) with a regimen consisting of Adriamycin, cyclophosphamide, capecitabine, and paclitaxel. The results showed a significant decrease in the PD-1^+^ T-cells population, but this reduction did not have a remarkable association with prognosis and complete pathological response (pCR) (86). A study on patients with stage II-III NSCLC compared two treated and non-treated groups based on receiving or not receiving NAC regimens including carboplatin plus paclitaxel or pemetrexed and cisplatin plus gemcitabine. Immunohistochemistry (IHC) analysis revealed a higher density of PD-1 expressing antigen-experienced and memory antigen-experienced cells (87). In a cohort study by Lo and colleagues on post-NAC tumor samples of 90 patients with high-grade serous carcinoma (HGSC) of the ovary, despite the rise seen in density of the favorable tumor-infiltrating T cells and B cells, no remarkable changes were seen in patients’ survivals after NAC with paclitaxel plus carboplatin. They hypothesized that this poor association could be attributable to the probable immunosuppressive effects of chemotherapy on the TME.

Assessment of the changes in the expression level of the inhibitory immune markers clarified that levels of IDO-1, FOXP3, and PD-L1 did not differ notably pre- and post-NAC. In contrast, PD-1 levels showed a considerable and significant increase in post-NAC samples compared with pre-NAC ones. This finding meant that an increase in the number of tumor-infiltrating lymphocytes (TILs) expressing PD-1 (as a co-inhibitory immune checkpoint) has occurred and neutralized the positive immune-stimulatory effects of the chemotherapy (88). Miyazaki et al. assessed the alterations in the expression of immune markers containing PD-1 and PD-L1 in initially and secondary resected samples of glioblastoma (GBM) from 16 patients who received chemotherapeutic agent temozolomide (TMZ) combined with fractioned radiotherapy (FRT) after the first surgery, before recurrent tumor surgery. IHC assays revealed significantly increased staining scores for CD3, CD8, and PD-1 in secondary resected specimens. Based on the PD-1 staining score, patients were categorized into low or high PD-1 score groups. Assessments to determine the prognostic value of PD-1 expression score in these two groups showed that a high PD-1 expression score was accompanied by longer progression-free survival (PFS), shorter survival after recurrence, and briefly poor prognosis (89). Considering what was mentioned above about the inhibitory role of the PD-1 pathway, which leads to T cell exhaustion and formation of an immunosuppressive context in the TME, and also paying attention to the predominance of the increasing pattern in PD-1 expression in the TME after chemo(radio)therapy in some solid tumors, it seems that combining these conditional treatments with immunotherapeutic agents inhibiting PD-1 specifically, may promote the efficacy of these anti-tumor approaches and improve the prognosis of many cancers.

## PD-L1

Programmed death-ligand 1 (PD-L1), also known as CD274 and B7 homolog 1 (B7-H1), is a member of the B7 family of type 1 transmembrane protein receptors. B7-H1 gene was discovered and cloned by Dong et al. 1999 (90), and its name changed to PD-L1 after recognizing its interaction with previously known PD-1 molecule (91). This protein is expressed in the many immune cells subtypes, including T cells, B cells, NK cells, Monocytes, and APCs, such as dendritic cells (DCs) and macrophages (**Table 1**) (53, 63). Expression of PD-L1 is increased following stimulation of some cell types by pro-inflammatory cytokines such as IFN-γ and IL-4 (68). As mentioned before, evidence indicates that activation of the PD-1/PD-L1 signaling pathway suppresses T cell-mediated immunologic responses in peripheral tissues and avoids effector T cells giving rise to tissue damage, the process described as immune “tolerance” (92, 93). Considering this crucial role of the PD-1/PD-L1 pathway, it is expected that cancerous cells use this property as an evasion mechanism halting the immune system’s anti-tumor function (92, 94). It has been proven that a wide variety of tumors upregulate the expression of PD-L1 on the surface of their cells as a mechanism to evade the immune system (95). Thus, inhibiting PD-L1 through designing and utilizing specified monoclonal antibodies (mAbs) has been widely brought into play in cancer immunotherapy in recent years. Some of these immune checkpoint inhibitors (ICI) like Durvalumab, Avelumab, and Atezolizumab are FDA-approved (**Table 1**) (51, 96, 97). A clinical trial study (NCT02639065) of Durvalumab on thirty-seven patients with esophageal cancer showed a relapse-free survival (RFS) rate of 73% (98). Similar to other immune markers and elements and as a consequence of alterations in the TME, several studies have demonstrated variations in PD-L1 expression patterns after traditional cancer treatments, including chemotherapy, radiotherapy, or a combination of them (**Table 1**). According to a systematic review by Van den Ende and colleagues, until January 2019, 48 studies had evaluated PD-L1 expression changes in response to common chemotherapeutic regimens, radiotherapy, or a combination of these approaches. Statistical analysis revealed that 30 articles reported higher expression of PD-L1 comparing pre-treatment and post-treatment specimens or comparing treated vs. untreated patients’ samples in cohorts. Just half of these increases were statistically significant. Among these studies are fluoropyrimidine-based neoadjuvant chemoradiotherapy of rectal cancer in 3 individual studies, two studies on ovarian cancer treated with carboplatin/paclitaxel regimen, two studies on head and neck squamous cell carcinoma with two different NCT regimens based on cisplatin or docetaxel, platinum, and fluorouracil and single studies on mesothelioma of the pleura, NSCLC, and basal cell carcinoma (BCC). Conversely, only eight studies reported decreases in the level of PD-L1 in post-treatment samples. Six studies demonstrated significant reductions, including single studies on FOLFOX-based treated rectal cancer, vinorelbine-based treated NSCLC, and nasopharyngeal cancer treated with chemoradiotherapy or radiotherapy alone (85). In a study by Lim et al. on 123 patients with rectal cancer, they compared pre- and post-NCT specimens immunohistochemically to assess the effects of NCT on the expression of PD-L1 and CD8+ TILs in the TME. Results demonstrated a rise in the expression levels of PD-L1 and the density of CD8^+^ TILs in post-NCT biopsies. Patients with high expression of PD-L1 pre- and post-NCT showed a lesser rise in CD8^+^ cells, and their overall survival and disease-free periods were significantly poorer. These findings may indicate the potentiality of applying combined methods such as simultaneous therapy with NCT and immune-checkpoint inhibitors (99). Ogura and colleagues did a similar study on 287 patients with rectal cancer evaluating PD-L1 expression and CD8^+^ cells density in the stromal and tumor compartments of the TME before and after chemoradiotherapy (CRT). This study showed an increase in PD-L1 expression on the stromal immune cells but not on the tumor cells. This finding was correlated with a high count of the tumor area’s CD8^+^ cells pre-CRT and high stromal density of CD8^+^ cells post-CRT (100). Song et al. carried out a study on 76 patients with squamous cell carcinoma (SCC) of the lung, comparing PD-L1 expression levels pre- and post-NCT with gemcitabine or Taxane plus platinum agent. Results demonstrated a significant up-regulation in PD-L1 expression post-NAC. PD-L1 positive patients had a poorer prognosis with shorter overall survivals (101). As mentioned before, some studies have paradoxically reported a reduction pattern in the expression of PD-L1 post-CRT. For instance, in a study by Zhang et al. on 109 patients with rectal cancer, the proportion of PD-L1^+^ TILs were significantly lower in post-NCT (FOLFOX with or without radiotherapy) specimens associated with poorer prognosis. The precise mechanism for this alteration was not found, and a probable unknown stimulatory role for the PD-1/PD-L1 signaling pathway was suggested as an underlying mechanism (102). The noticeable point is that the chemotherapeutic regimens used in these studies with paradoxical findings differed from each other. So, the kind of applied chemotherapeutic agents can be an impressive factor altering TME positively or negatively in the case of every single immune cell and immune marker such as PD-L1. However, further investigations are needed to assess and confirm this hypothesis.

## CTLA-4

Activation of T-cell is a relatively sophisticated process that needs more than one stimulatory signal. One of the components is the co-stimulatory signal induced by the interaction of B7-1 (CD80) or B7-2 (CD86) molecules on the APCs with the CD28 molecules on the T-cells, which gives rise to signaling within the T cells. The consequences of this signaling include the proliferation of the T-cells, improved survival and differentiation *via* synthesizing and secreting growth cytokines such as IL-2, overexpressing genes involved in cell survival, and improving energy metabolism (103).

CTLA-4 is a CD28 homolog with more affinity to the B7 molecule. Binding CTLA-4 to B7, opposite to what CD28 does, not only does not lead to a stimulatory signal but also produces a co-inhibitory signal that results in limited Il-2 production, restricted T-cell proliferation, and lower survival (**Table 1**) (104, 105). CTLA-4 eliminates B7-1 and B7-2 molecules from the membrane of APCs *via* trans-endocytosis and produces its function inhibitory effect through signaling independent mechanism (106). CD4^+^ Regulatory T cells (Treg) need CTLA-4’s appropriate function to establish and preserve immune tolerance. Blocking CTLA-4 leads to Treg dysfunction and leads to multi-organ autoimmunity (107, 108). Ipilimumab is an FDA-approved anti-CTLA-4 monoclonal antibody (mAb) from the IgG-1 subclass, and its effect on melanoma metastasis has been evaluated. However, some studies, including a clinical trial (NCT02279732) evaluating the effect of combination therapy of ipilimumab with chemotherapy in patients with squamous lung cancer, have demonstrated that adding this mAb to the chemotherapeutic regimen does not alter the median OS significantly (HR=0.91) (109). Tremelimumab is another mAb developed against CTLA-4 with the same binding affinity (**Table 1**) (110, 111). A clinical trial study (NCT01853618) in patients with HCC introduced tremelimumab as a potential novel treatment for advanced HCC (112). Two mechanisms have been suggested on how these mAbs work, one of them emphasizing the inhibitory effect of mAbs on CTLA-4, which leads to enhancement of the CD28/B7 binding. The other suggestion proposes that these mAbs exhaust the Tregs in the TME (113, 114).

Due to a systematic review, four studies involving two studies on rectal cancer, a study on breast cancer, and another single study on esophageal cancer have evaluated the changes in the expression level of CTLA-4 in the TME after CRT or NCT alone until January 2019. Only two of these mentioned studies, including one on rectal cancer patients and the other on breast cancer, had significant results, however, opposing alterations in CTLA-4 expression (85). Kaewkangsadan and colleagues designed and conducted a study on sixteen patients with large and locally advanced breast cancers (LLABCs). They used a chemotherapy regimen of adriamycin, cyclophosphamide, capecitabine, and paclitaxel as the NCT. Then they evaluated the alterations that occurred in the TME of specimens and the association of these changes with the prognosis of the disease by comparing pre- and post-therapy biopsies. Different types of TILs and immune markers were studied. The results demonstrated that NCT agents employed in the study maintained the CD8+ TILs population. A significant decrease was seen in the number of circulating and tumor-infiltrating FOXP3+ and stromal CTLA-4+ T cells. These changes reduce the secretion of inhibitory cytokines such as IL-10 and TGF-*β*. No changes happened in the population of intratumoral CD8^+^and CTLA4^+^ T cells. Also, the analysis showed that high levels of CTLA-4+ T cells in the stromal compartment were significantly associated with pCR. However, there was no similar relation between intratumoral CTLA4+ T cells and the pCR (86).

In another study, Zhang et al. assessed the effect of two different methods of neoadjuvant therapy on the TME cells and immune markers such as CTLA-4 on 109 patients with rectal cancer. A group of patients received the FOLFOX regimen as the NCT. The other group received neoadjuvant chemoradiotherapy (NACR) consisting of FOLFOX plus radiotherapy. Overall, the results clarified that the expression level of CTLA-4 in both groups was significantly higher post-neoadjuvant therapy. The NACR group showed higher levels of CTLA-4 expression compared with the other group in a meaningful manner. They attributed this finding to the immune system’s response to radiation exposure to avoid the autoimmunity caused by radiation. This study also showed a strong correlation between CTLA4+ and FOXP3+ TILs. It can be related to the increase in the number of Tregs in response to radiotherapy. Despite these changes, there was no significant relationship between CTLA-4 levels and the quality of response to the therapies (102). Overall, considering what was said above, only a few studies have evaluated the CTLA-4 expression levels alterations in response to chemo(radio)therapy till now. So, it seems that there is a need for more and more studies about this key immune checkpoint to help us make firm statements on how and by which mechanisms different types of neoadjuvant regimens change the expression of CTLA-4, what is the clinical significance of these patterns and their effect on the prognosis of various types of cancers and the overall survival (**Table 1**).

## LAG-3

Lymphocyte activating gene 3 (LAG-3), also known as CD233, is another immune checkpoint from the immunoglobulin superfamily. It was identified by Triebel and colleagues in 1990 (115). This molecule is expressed on TILs, NK cells, B cells, and DCs (116–119). LAG-3, with structural similarity and a close gene placement to the CD4 gene, has more affinity to binding MHC class II molecule (120). During the last years, several ligands have been introduced for LAG-3 as MHC-II, LSECtin, Galectin-3, and fibrinogen-like protein 1 (FGL1) (**Table 1**) (121–123). The detailed mechanisms of the LAG-3 function have not been known yet. However, this immune checkpoint exerts an inhibitory regulatory effect in activating T-cells that restrains autoimmunity and saves tissues (124). Co-expression of LAG-3 and PD-1 on TILs in the TME gives rise to T cell exhaustion and the consequent unlimited tumor growth (57). Some studies have demonstrated improvement in anti-tumor immunity by inhibiting the PD-L1 and LAG-3 simultaneously using bispecific antibodies (125). So, inhibiting LAG-3 enhances the immune system’s anti-tumor function by improving the effectiveness of other types of immunotherapy (126). Some mAbs have been developed against LAG-3 (**Table 1**) (127). These mAbs block the interaction between LAG-3 and MHC-II in the TME and improve the induction of apoptosis in the tumor cells. For example, LAG-3-Ig fusion proteins like IMP321 or eftilagimod alpha increase the expression of co-stimulatory molecules and IL-12 secretion that, finally enhances tumor immunity (78, 128). A clinical trial study (NCT00349934) in metastatic breast cancer patients demonstrated that a combination of eftilagimod alpha and paclitaxel empowered immune responses and doubled the tumor response rate (129). Relatimab, another LAG-3 blocking mAb, is currently being used widely in clinical trials, such as the study on metastatic ovarian cancer to improve the progression-free survival (PFS) of the patients (NCT04611126). Another Anti-LAG-3 mAb, Sym022, has been evaluated in some clinical trials, including a study on patients with locally advanced/unresectable or metastatic solid tumors or lymphomas (NCT03489369). Only a few studies have investigated the alterations in LAG-3 expression patterns in the TME post-NCT and its relationship with the disease prognosis (**Table 1**). Wang et al. studied the effect of NCT with an Anthracycline/Taxane-based regimen on the expression levels of LAG-3 and some other checkpoint molecules and their prognostic value in 148 patients with Triple-negative breast cancer (TNBC). Results of the study demonstrated an increase in expression levels of four molecules: CD8, PD-1, PD-L1, and LAG-3. A significant increase occurred in the LAG-3 levels post-NCT. There was also a significant correlation between high LAG-3, PD-1, and PD-L1 levels in pre-NCT biopsies. Nevertheless, high levels of LAG-3 on TILs in post-NCT samples demonstrated remarkable differences in nodal status and PD-l expression levels. At last high numbers of CD8+ TILs and nodal status were introduced as the definite factors altering the prognosis of tumor post-NCT. Also, high expression levels of LAG-3, particularly combined with high levels of PD-1, were other poor prognostic predictors (130). In another study, Bottai et al. assessed the TILs by evaluating the density of CD4+, CD8+, and FOXP3+ T cells. They also determined levels of expression of some immune checkpoints, including LAG-3 and PD-1, in the specimens of TNBCs from patients who underwent operation post-NCT. The results revealed that high quantities of stromal TILs were an independent good prognostic predictor correlated with high expression levels of the LAG-3 and PD-1. However, there was no significant association between these molecules’ expression and patients’ outcomes (131). The controversy between the results of the two mentioned studies may be attributable to the surgical intervention involved in the latter study or may be due to the tumor heterogeneity. These are just hypotheses and need more evaluation to be confirmed. Considering what we discussed above, we need more studies to evaluate the changes in LAG-3 expression patterns in response to NCT, determine the exact mechanisms of its action, and determine its effect on the prognosis across different cancers.

## B7-H4

B7-H4, as a member of the B7 family, is a transmembrane protein discovered by three different teams of researchers in 2003 (132–134). Also, Salceda and colleagues isolated the molecule again later in 2005. They researched to identify the overexpressed genes in cancers, focusing on gynecologic ones (135). There exists much inconsistency in the expression and distribution of B7-H4 across various types of tumor cells and normal cells (**Table 1**). Some studies have identified this molecule’s mRNA in different normal tissues in the human as the ovary, testis, pancreas, lung, spleen, and liver (136). However, the IHC studies for this molecule on normal tissues were negative, which indicates the firm translational control on this immune checkpoint. The same study demonstrated the expression of B7-H4 on a significant percentage of ovarian and lung tumor biopsies (133). Other studies also identified the molecule on uterus, colon, and breast tumors specimens. The intensity of the staining and expression was correlated with the cancer stage. Other cancers, such as gastric, kidney, and liver tumors, did not show similar results (137, 138). Expression levels of B7-H4 alter in a dynamic pattern along with the changes in the TME. High levels of Treg-induced production of some cytokines such as IL-6 and IL-10 by tumor-associated macrophages (TAMs) give rise to the overexpression of B7-H4 on the surface of TAMs. Some studies have shown a negative correlation between Treg count and the level of expression of B7-H4 on TAMs with the tumor prognosis (139–141). Many studies have suggested the inhibitory effect of B7-H4 on activated effector T cells *via* different mechanisms such as reduced IL-2 secretion that leads to diminished cell proliferation. Also, inducing cell cycle arrest is another mechanism (142). Several studies assessed the effects of B7-H4 expression on cancer cells *in vitro* and *in vivo*. The results clarified that B7-H4 empowers the tumors in many aspects, as preventing the apoptosis of the cancerous cells, augmenting proliferation and cell adhesion, and finally increasing the ability of migration, invasion, and metastasis (135, 143–146). B7-H4 overexpression in lung adenocarcinoma gives rise to an immunosuppressive TME (147). Until now, researchers have developed and used different antibodies against B7-H4 in several studies, including a clinical trial assessing the effect of an anti-B7-H4 drug, FPA150, in patients with advanced solid tumors (NCT03514121). (**Table 1**) (148, 149). Immune system augmenting effects such as increased IL-2 production reversed inhibitory effects of B7-H4 on effector T cells. It increased T cell proliferation, indicating the promising results of bringing these blocking antibodies into play (150). Only a few studies have evaluated the changes caused by conventional cancer therapies such as chemotherapy and radiotherapy in the expression level of B7-H4 in the TME (**Table 1**). Maskey et al. performed a study to evaluate the effect of NCT on TILs and B7-H4 expression in patients with gastric cancer. The other goal was to determine the cells and markers associated with the overall survival. They also evaluated their impact on the prognosis of the disease. To do this, they assessed and compared the expression of different subsets of TILs and the levels of B7-H4 molecule in two groups of patients with gastric cancer. One of these groups went under the NCT (NCT group) before the surgery, while the other did not (nNCT group). The regimen used for NCT was the FOLFOX regimen. The number of participants was 102. The results, achieved by the IHC analysis on the post-surgery biopsies, indicated that the NCT group had significantly lower levels of expression of B7-H4 molecule but higher levels of CD4^+^ and CD8^+^ TILs. More analysis demonstrated that NCT alone had no significant effect on the overall survival (OS). However, patients with lower expression of B7-H4 in the NCT group had significantly higher OS. So, the level of expression of B7-H4 is associated with the prognosis of the disease in patients with gastric cancer. However, TILs levels do not correlate with the disease prognosis (151). It seems that we need more research across different types of tumors to assess the exact effects of adjuvant therapies on the expression of B7-H4 in the TME and to determine its effect on the response to therapies. Also, determining the relationship between the expression of B7-H4 and disease prognosis is very crucial.

## The role of personalized medicine in combination therapy

Despite the wide use of the various kinds of targeted cancer therapies, such as immunotherapy, and also their combination with traditional ones, including chemotherapy, radiotherapy, and surgery, a remarkable proportion of the patients getting these therapies do not achieve the optimal cure, i.e., they show full resistance at the first steps or face the tumor relapse after the primary success (152). Many studies have demonstrated that the underlying etiology of this failure is the intra-population diversities, including their specific genetic composition that gives rise to heterogeneities in their “omics” data, besides the environmental factors. Omics, including the terms such as transcriptomic and proteomic, are indeed the connectors of the genotype of each individual to his phenotype. For example, a transcriptome is the whole mRNA of a subject or specimen. Methods such as microarray analysis and RNA sequencing techniques help us provide the transcriptomic information we need about the level of expression of different biomarkers and proteins, such as immune checkpoints. Also, the proteome is the entire protein expressed by a cell or a tissue, such as a tumor sample. Tools like mass spectrometry provide proteomic data about the characteristics of the proteins, such as expression amounts, post-translational alterations, cellular sites, and types of interactions between different proteins (153). Metabolomics is about recognizing and analyzing the metabolites, i.e., the mid-level molecules produced by the metabolic reactions. Metabolites are affected by genetic and environmental factors simultaneously, so a complete analysis of them can enlighten the specific response of an individual to a drug (154). The different omics techniques mentioned above are, in fact, the primary data collecting tools for a relatively new approach to cancer treatment, i.e., “precision medicine” or “personalized medicine.” The growing field of personalized medicine can be considered a revolution concerning cancer therapy, emphasizing designing and developing specific treatments for an individual or a group of patients based on data demonstrating their unique genetic, physiologic, and environmental features (155). The mentioned data can help us predict the response of different patients with diverse characteristics to a specific treatment shifting the trend of using generic medicine for all the patients of a particular disease to a specified and precise approach. The TME heterogeneity is one of the cardinal factors that bring about dissimilar responses in different individuals getting the same treatment, whether a single or a combination therapy (156). Cancer vaccines, mAbs (including ICIs), and CAR T-cells are among the personalized medicine-based therapies currently being used. As we mentioned in the previous sections, immune checkpoints blocking agents or ICIs are currently among the widely used drugs in both immunotherapeutic strategies and combination therapies. From the perspective of personalized medicine, to get more benefit from using ICIs and other target therapies, we need data and diagnostics to assess the possibility of a suitable response from a particular individual’s tumor. So, we need more studies to clarify the details of the immunologic pathways in which the immune checkpoints are involved, intending to recognize and introduce more biomarkers and other diagnostic elements that can help us anticipate a patient’s response to a particular drug or treatment (16). Only a few studies exist about personalizing traditional cancer therapies while personalizing these methods seems necessary due to their role and importance in different therapeutic approaches, such as in developing combination therapies. Wang et al. suggested that performing an appropriate diagnostic process before therapy can help us execute personalized cancer chemotherapy (157). Identifying biomarkers using “omics” technologies, especially proteomics, can be useful in evaluating the possibility of good responses to chemotherapy. Culturing a patient’s cancer cells to determine drug sensitivity is another method for assessing the probability of favorable responses to chemotherapeutic agents (158).

The main concept of this paper, i.e., evaluation of the alterations in immune checkpoint molecules’ expression patterns in response to chemo/radiotherapy, is somehow related to personalized medicine. Considering what we mentioned about the TME features and its role in anti-tumor immunity, also its changes in response to traditional adjuvant therapies, including the alterations in immune checkpoints expressions, and paying attention to what we mentioned about personalized medicine in this part, it seems logical to involve the patient(s) individual features, such as omics and physiologic characteristics in designing more specialized and effective combination therapies for various types of cancers.

## microRNA’s targeting of IC

**Figure 3:**
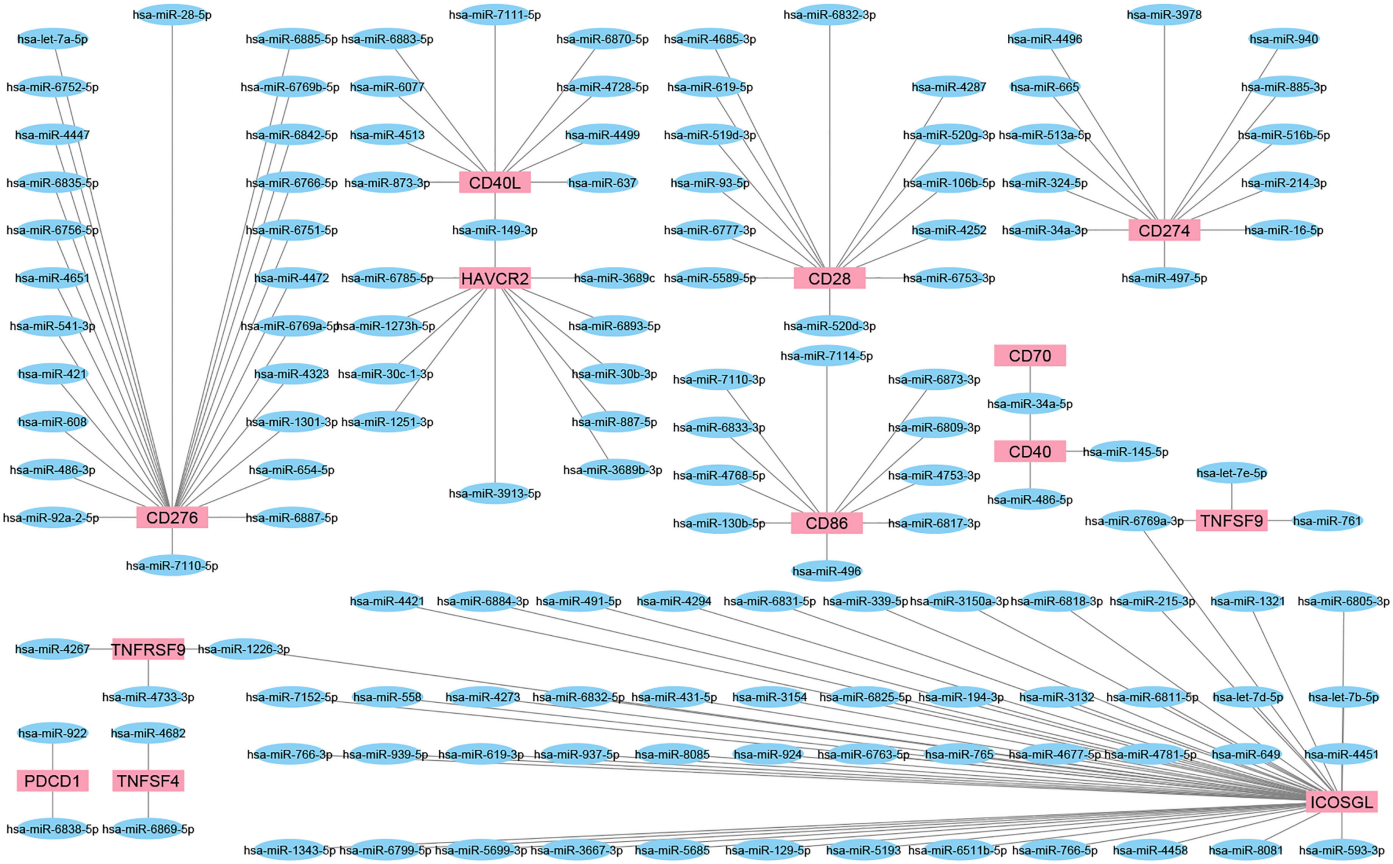
The interaction network of miRNAs and IC. These network shows the predicted interaction between some ICs and miRNAs. Targets or so-called IC are shown as red rectangles and predicted miRNAs as blue ellipses. Most miRNAs could target a single IC; however, miR-149-3p, miR-34a-5p, miR-6769a-3p, and miR-1226-3p could interact with more than one IC.

We employed a miRNA target prediction approach to consider the involvement of miRNAs during IC targeting and modulation of predicted miRNAs upon chemotherapy or radiotherapy. miRWalk v.3 was used to predict miRNAs with the ability to target IC (159). Also, the miRTarBase database of experimentally validated miRNA-gene targeting was employed to confirm the predicted interactions (160) (**Figure 3**). Then, the alteration of resultant miRNAs was considered by pieces of literature review. It is found that miR-194-3p is significantly down-regulated in docetaxel-resistant colon cancer cells. In addition, over-expressed miR-194-3p could promote SW620/docetaxel and SW480/docetaxel apoptosis and improve their docetaxel sensitivities. In addition, over-expressed miR-194-3p promoted docetaxel sensitivity of colon cancer cells by negatively regulating KLK10 (161). Overall, it was predicted that miR-193-3p could target PD-L1 and be involved in the activity of docetaxel. The results of a recent study highlighted the tumor suppressor roles of miRNA-486-5p mimic in bladder cancer carcinogenesis, identifying miRNA-486-5p mimic as an important therapeutic target in bladder cancer. Also, the results revealed that miRNA-486-5p mimic could increase cisplatin sensitivity in different bladder cancer cell lines and provide a better outcome for chemotherapy with cisplatin (162). A study conducted by Jin et al. showed the downregulation of miR-486-5p in nonsmall-cell lung cancer tissues compared with normal lung tissues and lower levels of miR-486-5p indicated a poorer prognosis for patients with nonsmall-cell lung cancer in terms of overall survival. Furthermore, this study demonstrated that miR-486-5p increased the sensitivity of A549 cells to cisplatin and inhibited EMT by directly targeting TWF1 (163). Also, it was predicted that miR-486-5p could target CD40 and involved in the activity of cisplatin. miR-761 expression is negatively associated with the expression of FOXM1 in colorectal cancer tissues. Elevated expression of FOXM1 suppressed the sensitivity of miR-761-overexpressing HT29 cells to 5-FU. It is also indicated that FOXM1 overexpression promoted cell proliferation, cycle, and invasion of miR-761-overexpressing HT29 cells. These data suggested that miR-761 played a tumor suppressor miRNA in colorectal cancer progression, and reduced miR-761 expression might be a major mechanism for 5-FU resistance in the colorectal cancer cell (164). Besides, it was predicted that miR-761 could target CD137L and be involved in the activity of 5-FU. A recent study indicated that miR-93-5p reduces the proliferation and migratory capacity of breast cancer cells and increases the ratio of apoptotic cells. Increasing apoptosis by overexpression of miR-93-5p may increase radiosensitivity in breast cancer cells (165). In addition, it was predicted that miR-93-5p could target CD28 and be involved in the activity of radiotherapy. It is demonstrated that CARM1 is highly expressed in cervical cancer tissues and radio-resistant cervical cancer cells, while miR-16-5p expression is low.

Under irradiation, up-regulation of CARM1 can induce radiotherapy resistance of cervical cancer cells, while overexpression of miR-16-5p or CARM1 knockdown could inhibit the survival of CC cell and induced apoptosis. Therefore, CARM1 was verified as a target for miR-16-5p. Besides, up-regulation of CARM1 reversed the increase in radiosensitivity induced by miR-16-5p (166). In addition, it was predicted that miR-16-5p could target PD-L1 and involved in the activity of radiotherapy. Additionally, it is reported that ionizing radiation (IR) exposure impaired lung cancer cell viability and found that miR-339-5p is a novel IR-inducible miRNA. Overexpression of miR-339-5p enhanced radiosensitivity of A549 and H460 cells by inhibiting cell viability, increasing apoptosis, inducing cell cycle arrest, and suppressing cell proliferation. Further exploration validated that miR-339-5p can target phosphatases of regenerating liver-1 (PRL-1) in lung cancer cells (167). Besides, it was predicted that miR-339-5p could target ICOSL and involved in the activity of radiotherapy.

## Conclusion and future perspective

Over past decades, significant advances have been made in cancer treatment, such as immunotherapeutic approaches using ICIs. Despite this, only limited types of cancers and a limited number of patients take advantage of immunotherapy. To design more effective therapies, we need to recognize the changes occurring in the TME across different types of tumors in response to various treatments. In this study, we have reviewed the alterations in the expression patterns of well-known and relatively newly found immune checkpoints during various RCTs. Different studies demonstrate that many factors such as the type of tumor and the type of chemo(radio)therapeutic regimen can influence the immune checkpoint expression patterns. Evaluating the results of different studies showed that the changes in immune markers in the TME are dependent on the number of TILs present in the tumor to a great degree. Tumors with higher numbers of TILs become more active, expressing higher levels of immune molecules and neoantigens post-NCT. These tumors are usually referred to as hot tumors, such as ovarian, rectal, and pancreatic tumors, and seem to be more promising targets for immunotherapy post-NCT and surgery. There have been many studies on some of the immune checkpoints, such as PD-L1. Most of them have shown the upregulation of this molecule following NCT. However, there are only a few studies with inconsistent results about other immune checkpoints, such as CTLA-4, LAG-3, and B7-H4. It indicates a need for designing more comprehensive studies. A remarkable number of studies showed that some changes in the immune checkpoints’ expression patterns were associated with the prognosis of the disease. It shows the necessity of becoming more knowledgeable about the alterations happening in the TME and its different elements, such as the checkpoint molecules in response to different chemo(radio)therapeutic approaches. Also, the number of studies about the effect of chemo(radio)therapeutic neoadjuvant therapies on the expression patterns of co-stimulatory immune checkpoints in the TME seems insufficient. So, there is a need for more studies to prepare the field for designing better combination therapies consistent with the concept of personalized medicine. The other point not noted in the mentioned studies is the link and association between the expression patterns of different immune checkpoints. Some studies have demonstrated a correlation and association between immune checkpoints’ genes originating from the “co-expression gene networks” (168, 169). This fact indeed gives us some clues which guide us toward considering immune checkpoints as a connected network rather than single independent genes. However, almost all the studies reviewed in this paper had not noticed this determining point. So, we must pay attention to the co-expression gene networks, i.e., the linkage between immune checkpoints’ genes, that brings about their probable simultaneous and correlated up-regulation or down-regulation in the TME before and after interventions such as chemo/radiotherapy to develop more effective and promising combination therapies.

## Author contributions

HH, the first author of the manuscript, searched, collected papers, and wrote the initial version of the manuscript. ZA, AD, and ADu left comments, and revised the manuscript. NR, SN, and OB contributed to manuscript preparation. AB provided figures. NS and BB the corresponding author of the manuscript supervised the project and also contributed to the revising of the main text of the manuscript. All authors have read and agreed to the published version of the manuscript.

## Conflict of interest

The authors declare that the research was conducted in the absence of any commercial or financial relationships that could be construed as a potential conflict of interest.

## Publisher’s note

All claims expressed in this article are solely those of the authors and do not necessarily represent those of their affiliated organizations, or those of the publisher, the editors and the reviewers. Any product that may be evaluated in this article, or claim that may be made by its manufacturer, is not guaranteed or endorsed by the publisher.
